# Damaged Lives and Assurance Medicine

**Published:** 1895-05-04

**Authors:** 


### The Hospital

AN INSTITUTIONAL JOURNAL OF

The Medical Sciences and Hospital Administration,

WITH

AN EXTRA NURSING SUPPLEMENT.

VOL. XVIII.—No. 449.] [MAY 4, 1895.

### DAMAGED LIVES AND ASSURANCE MEDICINE.

"DAMAGED lives," in large towns, and among men of thirty-five and upwards, are the rule and not the exception. No doubt this proposition will be strenuously denied, even by some assurance medical officers, and much more by those practitioners whose experience of "town practice" is very limited. Nevertheless, we repeat the proposition, and are prepared to defend it against all comers.

It requires no elaborate process of reasoning to convince the unprejudiced man of average intelligence that the case is as here stated. Take a typical countryman of the better classes, whose physique is good, whose means are adequate, whose mode of life is simple and unexciting, and compare him, not with the average, but with one of the best specimens of town-bred and strenuously toiling men who are bearing the brunt of the business of the nineteenth century. Compare the two in appearance, in appetite and digestion, in sleep, in the power of enduring fatigue, in youthfulness of spirits, in the functional condition of the nervous system, the liver, lungs, kidneys, heart, and blood vessels. The countryman may represent the normal standard of healthy life. When town men are compared with that standard, and the comparison is made with adequate physiological insight and skill, more than half of all the inhabitants of our larger towns above thirty-five years of age must be held to be "damaged lives."

Life assurance offices are constantly assuring "damaged lives." More than half of all persons assured, when compared with a high standard, are "damaged" at the time of the payment of the first premium. In discussing the question of the assurance of damaged lives it is not a question of normally perfect lives as against those that are imperfect or damaged, but a question of what kind and degree of imperfection shall be held to necessitate exclusion, and what kind and degree shall justify inclusion, and on what terms.

Life assurance is one of the "resources of civilisation"; and from that point of view must it be considered. Especially may this be said of that form of assurance known as "endowment assurance." When a man of the middle class can afford to pay an annual premium which will result in the return to him, say at the age of sixty-five, of a sum of five or six thousand pounds, or of the payment of that sum to his relatives in the event of his death at any time between the disbursement of his first premium and the age of sixty-five, that man may ordinarily die, at any rate in moderate peace, immediately such an assurance is effected. In these days of diminishing incomes and increasing expenditure it is exceedingly difficult—and especially for professional men, such as clergymen, doctors, and lawyers—to save money in considerable sums. Endowment assurances, which provide for a man's family in the event of his early death, and for his own old age in the event of his survival to that period, are among the simplest, easiest, and most effective methods of saving against the inevitable "rainy day" which social economics have yet devised.

It is of the first importance, therefore, to almost all the members of the middle class, and to large numbers of the better paid working class, that life assurance offices should enlarge their boundaries in all directions, and instead of insisting upon the assurance of relatively perfect lives only, should do all that science and experience will warrant to provide assurance for "all sorts and conditions," even of "damaged lives."

The question in the long run is purely a medical one, and that is the *raison d'etre* of its discussion in these pages. A short time ago Dr. Hingston Fox read a paper on the subject before the members of the Assurance Medical Officers' Association. That was a step in the right direction; but if the assurance offices are alive to their own interests and are minded to serve the public in the most liberal and public-spirited way possible to them, they will not allow the facts and principles then brought to light to be buried in the archives of a comparatively new and obscure medical association.

In practice, and in order that life offices may push forward beyond the grooves of past experience, and recognise their true functions of being savings banks for the more responsible classes, two things are necessary to be done. The first is to provide more competent and judicial medical examinations for candidates for assurance. At the present time every considerable office has one or more chief medical officers, who are, as a rule, of a fair standard of physiological and medical competence. But large numbers of candidates never appear before such chief medical officers at all, but are examined hap-hazard by the first general practitioner the assurance agent or inspector can lay his hands upon. Now, from the point of view of the judicial assessments of the value of damaged lives, many general practitioners are of little if any more competency than the assurance inspectors who engage their services. For the just assessment of such lives men of the highest physiological and medical competence must be sought, and they must be adequately paid.

The other indispensable condition of the widely-increased assurance of damaged lives is insisted upon by Dr. Hingston Fox in his paper. It is this, that candidates for this class of assurance must be prepared to take some part of the risk of assurance upon their own shoulders. The usual method of dividing such risks is to ask the assurer to pay premiums upon a certain amount, and then for him to agree that his executors shall accept that amount with certain strictly defined deductions if he dies before a specified age. This is entirely fair, and if understood by the assuring public would be seen to be so. The whole subject is of great importance to the professional and middle classes generally, and is well worthy of some great effort of general instruction on the part of life offices themselves.

